# CABRA: Cluster and Annotate Blast Results Algorithm

**DOI:** 10.1186/s13104-016-2062-y

**Published:** 2016-04-30

**Authors:** Pablo Mier, Miguel A. Andrade-Navarro

**Affiliations:** Faculty of Biology, JGU Mainz, Gresemundweg, 2, 55128 Mainz, Germany; Institute of Molecular Biology, Ackermannweg 4, 55128 Mainz, Germany

**Keywords:** Clustering, BLAST search, Web tool, Computational biology

## Abstract

**Background:**

Basic local alignment search tool (BLAST) searches are frequently used to look for homologous sequences and to annotate a query protein, but the increasing size of protein databases makes it difficult to review all results from a similarity search.

**Findings:**

We developed a web tool called Cluster and Annotate Blast Results Algorithm (CABRA), which enables a rapid BLAST search in a variety of updated reference proteomes, and provides a new way to functionally evaluate the results by the subsequent clustering of the hits and annotation of the clusters. The tool can be accessed from the following web-resource: http://cbdm-01.zdv.uni-mainz.de/~munoz/CABRA.

**Conclusions:**

Cluster and Annotate Blast Results Algorithm simplifies the analysis of the results of a BLAST search by providing an overview of the result’s annotations organized in clusters that can be iteratively modified by the user.

## Background

Every day it is becoming more evident that we must change soon the way protein databases are used. The continuous increase in the number of sequences deposited in these databases offers valuable evolutionary information that can be used for the study of protein function, but also makes such analyses more complex.

The most extended way to functionally annotate a sequence is using basic local alignment search tool (BLAST) searches [1] and the orthology information drawn from them. These are usually carried out in web servers like National Center for Biotechnology Information (NCBI)’s, whose output is limited to display the alignments of 20,000 hits. Even without this limit, it is clear that reviewing hundreds or thousands of results is a tough and time-consuming task. However, ideally, the variability given by all hits should be taken into account to improve the annotation process of the sequence of interest.

One approach to overcome this problem and get compressed results for a BLAST search is to cluster the database prior to the search, as in UniRef [2] or FastaHerder2 [3]. But a limitation in running sequence similarity searches against cluster representative sequences is that it is assumed that similarity to the cluster representatives implies similarity to the respective clustered sequences: this is not necessarily the case. More importantly, if the level of clustering is not appropriate for the description of the protein families of interest, which is generally the case, the user has to repeat the search with different clustered datasets, which is time consuming. In the end, the desired level of clustering might not exist in these pre-clustered databases. It seems more logical to cluster the results of a sequence similarity search in the databases, and not the entire databases, allowing the user to try different clustering cutoffs after examination of initial results in an iterative fashion. Cluster and Annotate Blast Results Algorithm (CABRA) [4] uses this different approach, as it clusters the results of a search in a selected database, and not the database itself. In addition, CABRA annotates the clusters, allowing the user to review the results’ annotations in a blink. Users can easily modify the parameters and repeat the clustering at will, without the need to repeat the search.

## Implementation

BLAST searches are performed using the BLAST 2.2.31 package [5] with e-value threshold = 1e−05, low complexity filter off, BLOSUM62 matrix and the curated protein database SwissProt (version 2015_10) as default parameters. Apart from SwissProt, there are also 578 available reference proteomes from different taxonomic groups that can be used as database (see full list at http://cbdm-01.zdv.uni-mainz.de/~munoz/CABRA/info/databases.html). They were all downloaded on 4/11/15 from the UniProt FTP site (ftp.uniprot.org/pub/databases/uniprot) [6].

The clustering of the BLAST results is done on-the-fly using the FastaHerder2 standalone algorithm (http://cbdm-01.zdv.uni-mainz.de/~munoz/fh2/) [3], with default parameters. CABRA uses annotations extracted from UniProt (release 2015_10) [6], such as the domain architecture, related protein data bank (PDB) entries and taxonomy information for each protein.

## Findings

### CABRA’s pipeline and its implementation in a user-friendly web tool

**Fig. 1:**
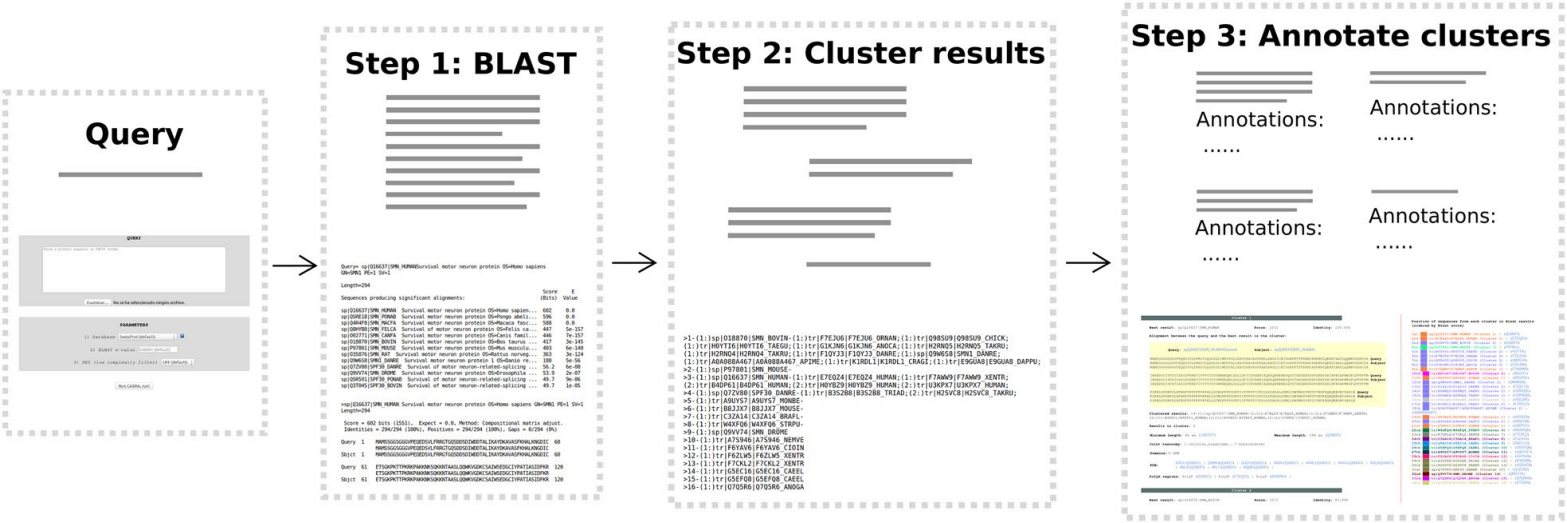
CABRA pipeline. Steps followed by CABRA. See text for details

CABRA consists of different modules implemented in Perl that work in three steps (Fig. 1) [4]. The first step requires a protein sequence in FASTA format, which is BLASTed against the sequence database. The database is composed of proteins from a selected set of complete reference proteomes and the SwissProt database. The amino acid sequences from the hits are retrieved and clustered in a second step, using a modified version of the FastaHerder2 clustering algorithm [3]. CABRA uses a greedy algorithm to cluster the sequences in two distinct steps. First the sequences are sorted by length, the longest one is taken as cluster’s representative and other sequences are clustered with it if they (1) have a similar length (less than 32 amino acids of difference following optimized values from the tool FastaHerder [7]) and (2) a level of identity above a threshold, which is the largest of either a length dependent one based on the Rost curve or a fixed value (53 %) (as used in FastaHerder2 [3]). The clustered sequences are removed from the dataset and the procedure is iterated until no sequences remain.

In a second step, singletons (usually sequence fragments or splice variants) are added to the existing clusters if their sequence is above the identity threshold to the cluster’s representative irrespective of length differences. Sequences clustered in this step are marked with a “2:” in the outputs. The default value of 53 %, applied in both steps, can be modified by the user, but the Rost curve always remains as minimum identity limit and cannot be overridden.

The clustering of the BLAST results allows the user to visually detect and classify functionally important hits, sometimes not a trivial task. Even fragments very similar to the query sequence can have a low BLAST score due to their length, and therefore they would not be within the top results in a usual similarity search. The same can happen with orthologous sequences from distant species, which may be less similar to the query than the query’s paralogs. The clustering step is able to cluster them with one or more of the top results, not letting them pass unnoticed.

In a last step, CABRA annotates the formed clusters to give the user information about the proteins in them, such as their PDB entries (if any), domain architecture, length of the proteins, and their common taxonomy (joint taxonomy). The annotations are obtained from the individual UniProt entries of the proteins in each cluster. Both the BLAST report and the set of clusters are provided. To ease the visualization of the BLAST report, we provide a color-coded simplification of the results based on the cluster components. For each protein, its percent identity compared to the query is also shown.

**Fig. 2:**
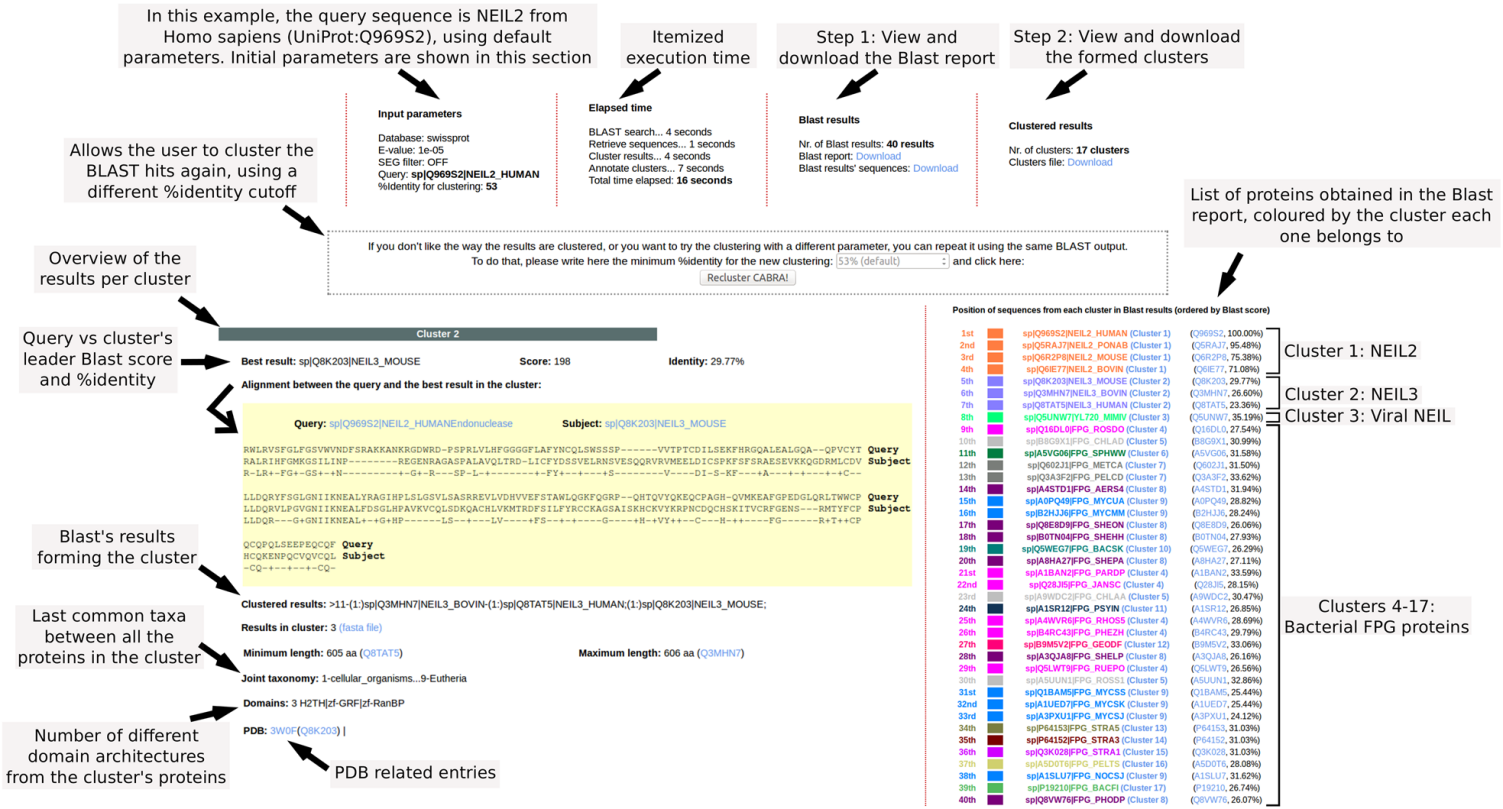
Illustrative explanation of the result’s layout after the execution of the CABRA web tool. *Top section* overview of the search; *bottom right section*, list of proteins from the BLAST report; and *bottom left section*, reviewed information per cluster. Only the information for one cluster is shown

The output of the web tool is distributed in three main sections (Fig. 2): the top one, in which an overview of the search is shown (input parameters, elapsed time, and downloadable results); the bottom right section, where the list of proteins obtained in the BLAST report is displayed, colored based on the cluster each one belongs to; and the bottom left section, in which each cluster’s information is reviewed. The visualization of the overall annotations from a cluster provides a more understandable representation of the BLAST output; for example, if each cluster reflects a specific domain architecture the results can be easily interpreted.

CABRA was developed to be used as a web tool, but a standalone version of it is also provided in the web tool’s homepage. Using the standalone CABRA the user can use his own database along with the query sequence.

### Example of the performance of CABRA in comparison to other web tools

To illustrate the performance of CABRA with an example, as well as its utility and improved output, we used as query sequence the human protein NEIL2 (UniProt:Q969S2), using default parameters. NEIL2 is an endonuclease VIII-like zinc-finger FPG-type protein with one H2TH domain involved in DNA repair [8]. In 16 s CABRA finds 40 BLAST hits and groups them in 17 clusters, two of them containing eukaryotic proteins and the rest with prokaryotic or viral proteins (Fig. 2). The first four BLAST hits form one cluster containing NEIL2 proteins with an H2TH domain, as expected. The next three hits are NEIL3 proteins, a paralog of NEIL2 that is restrained to a second cluster. These three proteins have a similar three-domain architecture, with the domains H2TH, zf-GRF and zf-RanBP. The third cluster contains only one viral endonuclease VIII-like zinc-finger FPG-type protein, similar to the human NEIL proteins [9]; as it is only 35.19 % identical to the human query protein, it was not clustered in neither the first nor the second cluster, but in a different one. Like the NEIL2 proteins, it also contains one H2TH domain. The remainder of the results are all bacterial FPG proteins with three domains (Fapy_DNA_glyco, H2TH, and zf-FPG_IleRS), and they are distributed in multiple clusters because their sequence similarity is below the minimum identity cutoff (the 53 % default value was used).

For comparison, a similar search in UniProt, using the UniProt web BLAST server (http://www.uniprot.org/blast/, query UniProt:Q969S2, database: UniProtKB/Swiss-Prot, e-value threshold = 0.0001) takes approximately the same time (around 15 s), but discriminating the orthologs of the query (NEIL2) from its paralogs (NEIL3) and from its ancestors (FPG) represents a daunting task. The functional and structural information that can be drawn from each protein group is different and therefore they would have to be looked upon separately. Using CABRA, the on-the-fly annotation of the clusters eases the interpretation of the results, identifying the FPG proteins as bacterial and the H2TH domain as the common link between all BLAST results, as described in the bibliography [10].

For comparison to using a pre-clustered database, we repeated the search using the same parameters as above, but against the UniRef50 as database. While the execution time is the same as in the previous searches, the results are much more dispersed in the clusters. For example, the NEIL2 proteins are present in at least two clusters; one of them contains the orthologs for human and *Pongo abelii*, while the other one has the mouse’s and cattle’s orthologs. These four proteins represent only one cluster in CABRA.

CABRA uses a pipeline that is based on clustering a set of sequences known to be similar to the query sequence; on the other hand, databases like UniRef [2] limit the search space by clustering the database, and then comparing the query to the cluster’s representatives. The computation of the clusters after getting the BLAST output has the clear advantage that the query sequence is compared to all of the results, and not just to the cluster’s representative, as in UniRef.

**Fig. 3:**
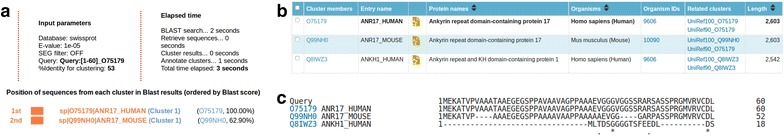
Comparison of results from CABRA and UniRef50. The N-terminal region (positions 1–60) of the human ANR17 protein (UniProt:O75179) was used as query in both searches. **a** Results obtained after the search in CABRA; two ANR17 proteins in one cluster. **b** The best result from a search in UniRef50 with SwissProt proteins, the UniRef50_O75179 cluster; two ANR17 and one ANKH1 proteins are part of the same cluster. **c** Multiple sequence alignment of the three proteins from the UniRef50_O75179 cluster plus the query sequence

To illustrate the differences between both strategies with an example, we searched both the UniRef50 database using the UniProt web server, and the CABRA web tool (database: SwissProt) using as query the N-terminal region (positions 1–60) of the human ANR17 protein (UniProt:O75179) with the same e-value threshold (0.0001) and the rest of the parameters as default. CABRA computed the search in 3 s, while the same task in UniProt took around 93 s. Only one cluster with two sequences was found by CABRA, containing proteins ANR17 from human (UniProt:O75179) and mouse (UniProt:Q99NH0) (Fig. 3a). On the other hand, the first result from the search in UniRef50 with SwissProt proteins (to make a fair comparison to our results from SwissProt) is the cluster UniRef50_O75179, formed by three SwissProt proteins (Fig. 3b). These proteins are the ones found by CABRA plus the human ANKH1 protein, although the latter does not contain an N-terminal region similar to the query (Fig. 3c). As shown in this example, it is more coherent to look for similar sequences to the query, and then to structure the results by clustering them, than to search in previously clustered databases.

**Fig. 4:**
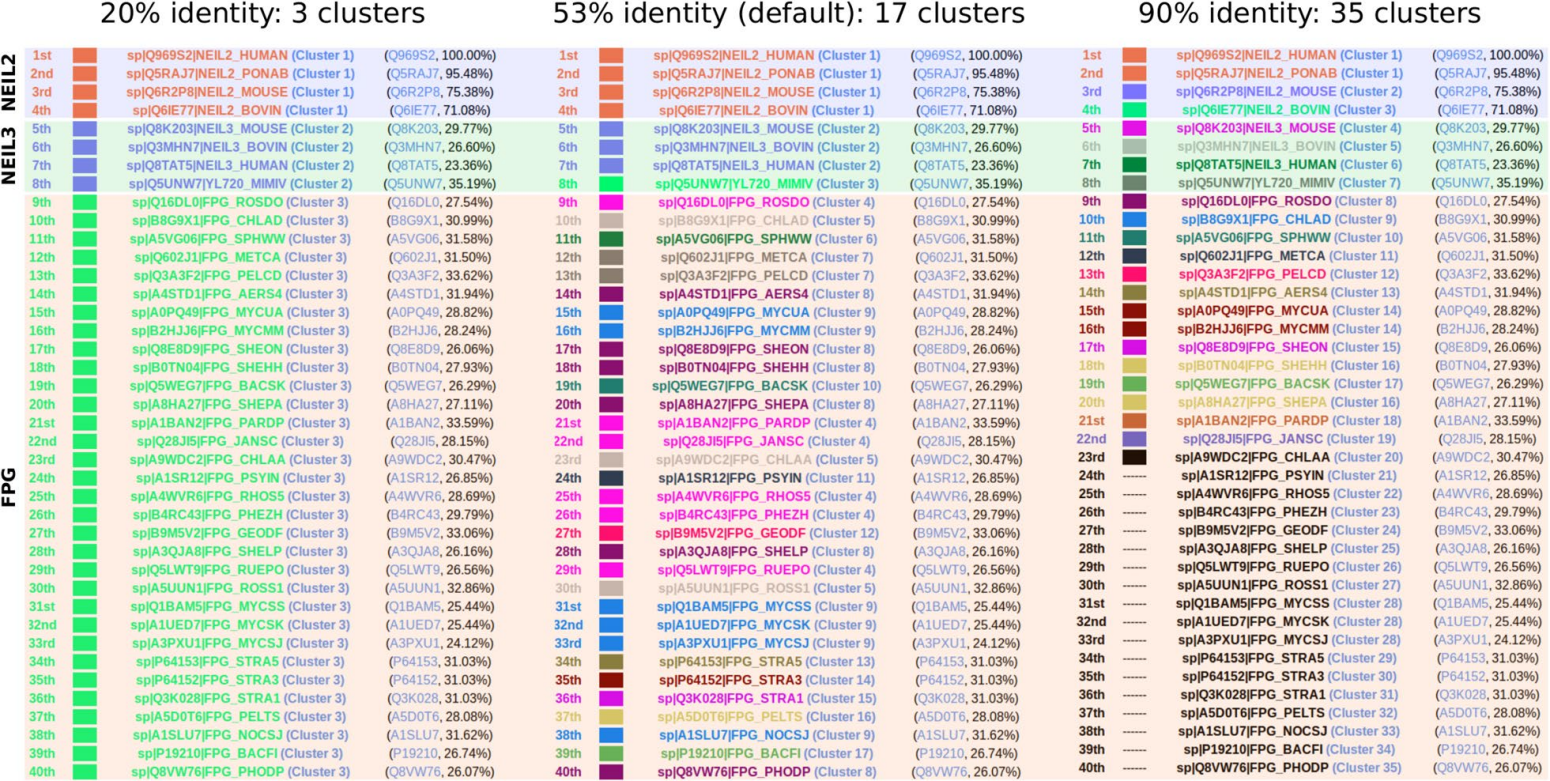
Overview of the hits obtained from a BLAST search clustered in CABRA using different identity cutoffs. Query: Q969S2, default parameters. BLAST hits clustered using three identity cutoffs: 20, 53 (default) and 90 %. Distinct *colors* to label the clusters are used up to the twentieth cluster

Although a similar approach as the one used by CABRA was being used by other tools like BlastGraph [11] and BLASTGrabber [12], the implementation in an easy-to-use web tool simplifies its execution and usage by different types of users. Furthermore, CABRA allows the customization of the clustering identity cutoff. After the results are shown in the output, the user is allowed to modify this parameter and CABRA clusters again the sequences obtained in the BLAST search. This feedback may help the user to find the clustering that better suits the results. For example, in the previous search (human NEIL2 protein as query (UniProt:Q969S2), default parameters), CABRA produced 17 clusters out of the initial 40 BLAST hits. When the identity cutoff is changed to 20 %, they are reduced to only three clusters: the first with NEIL2 proteins, the second with NEIL3 proteins plus the viral NEIL protein, and a third cluster with 32 bacterial FPG proteins (Fig. 4). On the other hand, when the identity cutoff is set to a stringent 90 %, almost no sequences are clustered together, and it results in 35 clusters. This example illustrates how the identity cutoff to cluster the sequences can modify the results, and the importance of looking for the optimal clustering for each protein family.

## Conclusions

The evaluation of a BLAST output is usually a tedious task, as one usually does not know how the results are distributed and has to go over all of them to study them thoroughly. CABRA provides a shortcut to this task, as its clustering of the hits allows for their quick classification. The separation of paralogs and fragments in different clusters eases the targeting of the cluster of interest, which, in addition, is annotated.

CABRA integrates in one pipeline the advantages of a BLAST search and of the clustering algorithm FastaHerder2 by annotating the clusters composed of BLAST hits. The simplification and annotation of the results from a typical similarity search, along with its easy usage and speed makes CABRA a suitable upgrade of today’s unidimensional similarity search tools. The possibility to tune the identity threshold to cluster the sequences similar to the query is also an improvement over the current clustering approaches.

We encourage all developers of sequence similarity search tools to follow our strategy as a simple way to overcome the problem of the increasing size of sequence databases with a solution that allows using all the sequence information from thousands of sequence similarity hits and facilitates their analysis.

## Abbreviations

CABRA: Cluster and Annotate Blast Results Algorithm
BLAST: basic local alignment search tool
NCBI: National Center for Biotechnology Information
PDB: protein data bank
